# An evolutionary parsimonious approach to estimate daily reference evapotranspiration

**DOI:** 10.1038/s41598-024-56770-3

**Published:** 2024-03-20

**Authors:** F. Javier Ruiz-Ortega, Eddie Clemente, Alicia Martínez-Rebollar, J. Jassón Flores-Prieto

**Affiliations:** 1 TecNM/Cenidet, Interior Internado Palmira S/N, Col. Palmira, CP 62490 Cuernavaca, Morelos Mexico; 2 TecNM/I.T. Torreón, Antigua Carretera Torreón - San Pedro KM 7.5, Ejido Ana, 27170 Torreón, COAH Mexico; 3 TecNM/I.T. Ensenada, Boulevard Tecnológico No. 150, Ex-Ejido Chapultepec, CP 22780 Ensenada, Baja California Norte Mexico

**Keywords:** Climate sciences, Hydrology

## Abstract

The reference evapotranspiration (ETo) is an essential component in hydrological and ecological processes. The objective of this research is to develop an explicit model to estimate ETo only using commonly measurable meteorological parameters such as relative humidity, air temperature, and wind speed, where the measurements corresponding to solar radiation are omitted. The model was generated using Genetic Programming (GP), evaluated, and validated with reference data ETo using FAO56-PM. This reference data was obtained from different climates (warm-temperate and arid-warm) and latitudes, acquired from CIMIS stations in the state of California, United States, and the El Porvenir station in the state of Coahuila, located in north-central Mexico. After applying the proposed methodology, a total of 3754 results were generated, demonstrating a significant improvement in the estimation of ETo compared to the Hargreaves–Samani model. A particularly noteworthy result revealed that our approach outperformed the Hargreaves–Samani model in the training phase by 27%, and in the testing phase by 16%, on average. In order to achieve a generalized model, a dataset encompassing meteorological stations in two different climates (warm-temperate and arid-warm) and various latitudes was utilized. The obtained outcome unveiled a highly effective model for estimating ETo in diverse climatic contexts, eliminating the need for local adjustments. This model significantly surpassed the Hargreaves–Samani model, exhibiting superior performance by 17% during the training phase and 18% during the testing phase. These results conclusively underscore the capability of our approach to provide more accurate and reliable ETo estimates. These results conclusively underscore the capability of our approach to provide more accurate and reliable ETo estimates. Finally, to validate the model, four different datasets with climates similar to those used for model creation (warm-temperate, warm-arid) and different latitudes were employed. The validation stage results clearly indicate the superiority of our reference evapotranspiration ETo11 model over the Hargreaves–Samani model by 51% in warm-temperate climates. For the dataset with arid-warm climate, our model continued to show satisfactory results, surpassing the Hargreaves–Samani model by 8%. GP emerges as an innovative and effective alternative for simplified model development. This approach introduces a novel paradigm that facilitates the efficient development of models, standing out for its simplicity and effectiveness in generating solutions.

## Introduction

Evapotranspiration (ET) is the combination of two separate processes, the first of which is the loss of water from the soil surface by evaporation and the second of which is crop transpiration. Evaporation and transpiration occur simultaneously, and there is no easy way to distinguish between these two phenomena. Since ET represents a vital component of the hydrological cycle, its precise estimation is necessary for the management of water resources, the hydrological balance of basins, and drainage system irrigation. The main climatic parameters that affect ET are solar radiation, temperature of the air, relative humidity, and wind speed.

In 1921, Cummings introduced an initial energy balance equation, which was later integrated by Penman in 1948 with a mass-transfer equation grounded in Dalton’s research, culminating in the formulation of the Penman equation. A crucial parameter in this equation was the Bowen ratio, first published in 1926. Subsequently, following Penman’s contributions, another significant advancement occurred in 1965 by Monteith. He refined Penman’s equation initially designed for a single leaf, giving rise to the Penman–Monteith model. This model, serving as the foundation for the FAO56-PM Reference Crop model, marked a pivotal milestone in the evolution of evaporation estimation techniques. McMahon and his collaborators provide a crucial discussion on the theory and fundamental definitions, along with a critical assessment of the models developed to date^1^.

The rate of evapotranspiration from a surface of reference, occurring without water constraints, is known as evapotranspiration of the reference crop, called ETo^2^.

However, it has significant limitations. Primarily, it requires a substantial amount of detailed data to obtain reliable results, which can be a challenge in situations of irregular data availability^3^. Additionally, the implementation of the model can be intricate, especially regarding the calculation of surface resistance, as indicated by previous studies^4^. These identified limitations underscore the imperative need to develop alternative models that streamline data collection and reduce computational complexity. On the other hand, the FAO recommends the Hargreaves–Samani model for places where only temperature data are available. Although it is true that the model only works with temperature differences, the calculation of daily ETo could be subject to errors due to the influence of the temperature range^5^.

A study conducted by^6^ provides a solution to the issue of the Hargreaves model by proposing an adjustment to suit the specific characteristics of semi-arid climates, typically Mediterranean, characterized by extremely hot and dry summers. This modification aims to enhance the accuracy of the original model by more appropriately accounting for the climatic peculiarities inherent in such environments, thereby enabling a more effective and reliable application in those contexts. However, it’s worth noting that the proposed solution is limited to a single climate type, and regional adjustments will be necessary for its implementation.

The estimation of ETo can be broadly grouped into three categories: (1) models entirely based on physical principles that incorporate the fundamentals of mass and energy conservation; (2) semi-physical models that specifically address mass or energy conservation; and (3) black-box models relying on artificial neural networks, empirical relationships, and fuzzy and genetic algorithms^7^. Currently, in addition to the mentioned models, remote sensing models have been developed and applied for ETo estimation. These models utilize data collected by remote sensors, such as satellite images, to assess and calculate ETo with greater precision^8^.

Some experts, as mentioned in the literature^7,9^, have delved into the application of genetic algorithms with the purpose of standardizing certain models to enhance estimation accuracy. These initiatives have placed particular emphasis on methods like Hargreaves and remote sensing technologies such as MODIS (Moderate Resolution Imaging Spectroradiometer). The primary goal of this approach is not only to refine the accuracy of estimations but also to ensure greater consistency and reliability in the results derived from these models and techniques. It is important to note that, despite these advancements, these models require specific adjustments to achieve a more precise adaptation to the particular conditions of the region under study. This recognition underscores the need to consider local factors in the application of these models to guarantee more accurate and contextually relevant estimations.

At present, evolutionary computation techniques such as GP, artificial neural networks, and neurofuzzy models have been used for modeling hydrological processes^10–14^. These techniques have the ability to model nonlinear complex processes and can estimate ETo accurately. However, their success depends on various factors such as the quantity and quality of the data, the selection of the model structure, and the required model parameters. In^13^, the authors estimated ETo using relative humidity and air temperature data. The authors used two strategies for handling data. The first strategy was clustering by climate type, and the second strategy used past meteorological data as input to the models. The techniques used in that study were a neural network and a support vector machine as well as six empirical equations; the latter showed the lowest performance. However, models generated through machine learning techniques such as artificial neural networks and support vector machines are perceived as black boxes due to their intrinsic complexity and internal algorithms involving multiple layers of interconnected nodes. This lack of transparency can lead to distrust and reluctance in their use, as they do not provide a clear explanation of the relationship between input variables and the obtained results. Additionally, the models were developed with clusters of data from weather stations with similar climates, which may result in inaccurate performance in regions with significantly different climatic conditions. ETo has also been addressed using genetic algorithms^11^ used daily climatic variables obtained from the California Irrigation Management Information System CIMIS^15^ of the Davis, Hasting, Suisun, Dixon, and Oakville stations. The authors evaluated the performance of genetic algorithms against various empirical models, such as Jensen–Haise, Hargreaves–Samani, Jones-Ritchie, the Turc method, and models based on solar radiation. However, despite the authors reporting good performance compared to other conventional methods, the new model uses the same input parameters as the FAO-PM reference model, limiting its applicability in areas where the necessary information is unavailable. Other authors, such as^16^, applied GP to estimate ETo in the Basque Country (northern Spain). The developed models used the meteorological parameters of relative humidity, solar radiation, air temperature, and wind speed. Although the models proposed by^11,16^ yield quite satisfactory results, it is important to note that they use the same parameters (air temperature, relative humidity, solar radiation, and wind speed) as the FAO-PM model. This similarity limits their implementation in locations where not all parameters are available. In^17^, the authors compared artificial neural networks using the standard Penman‒Monteith method. They worked with two datasets obtained from the Davis station of CIMIS. The variables used were solar radiation, maximum temperature, minimum temperature, maximum relative humidity, minimum relative humidity, wind speed and ETo obtained with the Penman‒Monteith standard method as the objective value. The results show that the artificial neural network model can be trained locally to predict ETo values better than the standard Penman‒Monteith method. However, despite the authors reporting positive performance, the new model uses the same input parameters as the Penman–Monteith reference model, limiting its applicability in areas where necessary information is not available. Additionally, being trained locally implies that, for use in another region, it will need to undergo a new training process.

Random forest models and generalized regression neural networks were used by^18^ to estimate ETo for the period 2009–2014 at two meteorological stations in China. The results show that the random forest model and the generalized regression network can be successfully applied to accurately estimate daily ETo. However, the random forest model may perform slightly better than the generalized regression neural network in estimating daily ETo. The presented models exhibit favorable performances; however, they are characterized as black-box models. Furthermore, it is worth noting that they were exclusively trained with a single type of climate, implying that if they are to be used in a region with different climatic conditions, they will need to undergo a new training process tailored to the specific conditions of the location.

The research conducted by^19^ compared different machine learning techniques for developing ETo models, achieving accurate results. On the other hand, the best-performing model presented involves variable net radiation, which often remains unavailable, limiting its implementation. Similarly, the models were generated for a humid climate. The extreme support machine in combination with genetic algorithms was employed by^20^ to develop models for estimating ETo. They found that the combination of these two techniques yielded accurate results using input variables such as T_max_, T_min_, u_2_, RH, and Rn/Rs, as well as T_max_, T_min_, and Rn/Rs. Nonetheless, these models were tailored for subtropical and plateau mountainous monsoon climates, and they utilize variables that are frequently unavailable, Such as solar radiation. Furthermore, these models are considered black-box models.

The models proposed by^19,20^ stand out for their higher accuracy compared to conventional models; however, their use of variables that are sometimes challenging to obtain, such as solar radiation, poses practical challenges. Additionally, these models were specifically designed for a particular climate, and their techniques, considered “black box,” present aforementioned disadvantages. The limitation in variable availability and climatic specialization may restrict the applicability of these models in different conditions or locations, emphasizing the importance of considering these limitations when assessing their suitability and applicability in broader contexts.

While some techniques found in the literature displayed in Table 1 demonstrate an accurate estimation of ETo, they face the challenge of being considered “black boxes,” complicating the interpretation of their results^13,19^. Furthermore, the findings presented involve the use of variables such as solar radiation and net radiation, the acquisition of which is hindered by the high costs associated with the necessary measurement equipment^11,16^. Additionally, a significant portion of the models has been designed to adapt to specific climates, posing complications in terms of generalization as they require a new training process when applied to climates different from those used during their development^19^. These challenges underscore the need to address the interpretability of the employed techniques and to develop more adaptable models that consider climatic variability to enhance their applicability in diverse contexts.

**Table 1:** Summary of the different articles addressing the issue of evapotranspiration.

Title	Parameters	Technology	Limitations
Evolutionary algorithm for reference evapotranspiration analysis^9^	Evapotranspiration one day ago, Evapotranspiration two days ago, Maximum and minimum temperatures, Hours of sunlight, Vapor pressure, Maximum and minimum relative humidity, Wind speed	Genetic Programming	Adapted to a single climate
Genetic Programming-Based Empirical Model for Daily Reference Evapotranspiration Estimation^10^	Solar radiation, Average temperature, Average relative humidity, Wind speed	Genetic Programming	Uses the same variables as the FAO-PM reference model
Generalizability of Gene Expression Programming-based approaches for estimating daily reference evapotranspiration in coastal stations of Iran^11^	Maximum and minimum temperatures, Maximum and minimum relative humidity, Solar radiation, Wind speed	Linear Genetic Programming	Uses the same variables as the FAO-PM reference model
Estimation of reference evapotranspiration in brazil with limited meteorological data using ann and svm–a new approach^12^	Average temperature, Average relative humidity	Support Vector Machine and Artificial Neural Networks	The techniques used are considered black-box type, which limits their analysis
Forecasting of Reference Evapotranspiration by Artificial Neural Networks^13^	Average temperature, Average relative humidity, Wind speed, Sunshine hours	Artificial Neural Networks	The techniques used are considered black-box type, which limits their analysis
Daily reference evapotranspiration modeling by using genetic programming approach in the Basque Country (Northern Spain)^15^	Average temperature, Average relative humidity, Wind speed, Solar radiation	Linear Genetic Programming	Uses the same varia-bles as the FAO-PM reference model
Estimating evapotranspiration using artificial neural network^16^	Solar radiation, Maximum and minimum temperatures, Maximum and minimum relative humidity, Wind speed	Artificial Neural Networks	The techniques used are considered black-box type, which limits their analysis
Evaluation of random forests and generalized regression neural networks for daily reference evapotranspiration modeling^17^	Solar radiation, Maximum and minimum temperatures, Maximum and minimum relative humidity, Wind speed	Random Forests and Artificial Neural Networks	The techniques used are considered black-box type, which limits their analysis
Evapotranspiration evaluation models based on machine learning algorithms—A comparative study^18^	Net solar radiation, Sensible heat flux, Soil moisture content, Wind speed, Average relative humidity, Mean temperature	M5P Regression Tree, Bagging, Random Forest and Support Vector Regression	The techniques used are considered black-box type, which limits their analysis
Genetic Algorithm-Optimized Extreme Learning Machine Model for Estimating Daily Reference Evapotranspiration in Southwest China^19^	Solar radiation, Net radiation, Maximum and minimum temperatures, Maximum and minimum relative humidity, Wind speed	Extreme Support Vector Machine with Genetic Algorithms	The techniques used are considered black-box type, which limits their analysis
Evaluation of Variable-Infiltration Capacity Model and MODIS-Terra Satellite-Derived Grid-Scale Evapotranspiration Estimates in a River Basin with Tropical Monsoon-Type Climatology^7^	Solar radiation, Maximum and minimum temperatures, Maximum and minimum relative humidity, Wind speed	MODIS, VIC_3L adjusts MODIS with Genetic Algorithms	Standardizes the parameters with genetic algorithms
Modelling the dynamics of evapotranspiration using Variable Infiltration Capacity model and regionally calibrated Hargreaves approach^3^	Solar radiation, Maximum and minimum temperatures, Maximum and minimum relative humidity, Wind speed	Genetic Algorithms	Adjusts the Hargreaves model with genetic algorithms

As evidenced in Table 1, the majority of the proposals under analysis share similarities in terms of the input variables used, aligning closely with the FAO-PM reference model. These proposals mostly adopt techniques considered as “black box.” However, an additional group of approaches stands out, aiming for the standardization of models such as Hargreaves and remote sensing techniques like MODIS through adjustments, employing genetic algorithms or kriging interpolation. It is important to note that some of these proposals are limited to the use of a single climatic dataset during their evaluation.

Within the scope of our research, we aim to develop an explicit model for easy evaluation. Our proposal is based on the use of commonly measurable variables, such as temperature, relative humidity, and wind speed. The primary objective is to achieve a precise approximation to the FAO-PM reference model. By opting for widely recorded variables, our intention is not only to simplify the model evaluation but also to enhance its practical applicability in environments where conventional meteorological data are available. This approach aims to contribute to the generation of more accessible and effective models in the field of evapotranspiration estimation.

In contrast, techniques like GP, by generating explicit models, offer advantages in terms of interpretability, facilitating the understanding, validation, and debugging of the model. The generalization capability and the incorporation of expert knowledge reinforce the utility of explicit models, highlighting their applicability in environments requiring a deep understanding of the domain. In this context, we have conceived a generalized and explicit model, enriched with expert knowledge in its formulation. This innovative approach utilizes easily measurable parameters such as temperature, relative humidity, and wind speed. It stands out for its ability to make accurate ETo estimations, surpassing the Hargreaves–Samani model. Furthermore, its versatility is evident in its applicability to two different climates: arid-warm and warm-temperate. This model not only optimizes result interpretation but also emerges as a valuable and adaptable tool for various climatic conditions, enhancing its utility in heterogeneous environments.

GP has emerged as an evolution of traditional genetic algorithms, maintaining the same principle of natural selection. What is now intended is to solve problems through the induction of programs and algorithms. It is in this possibility where all the potential of GP resides, as it allows the automated development of programs, understood in a broad sense, that is, as algorithms in which, based on a series of inputs, a series of outputs is generated. In this way, for example, a mathematical equation could be induced using GP.

Throughout history, extensive research has been conducted with the purpose of estimating Reference ETo using standard meteorological data. In this context, sustained efforts have been focused on reducing the number of variables and, consequently, the meteorological data required for the estimation of ETo^1^.

In our research, the FAO Penman‒Monteith method (FAO56-PM) is considered to be the knowledge expert since it is based on robust physical bases and explicitly incorporates physiological parameters and aerodynamics. The objective of this research is to generate an explicit model to estimate the ETo in at least two different climates without local adjustment using meteorological variables such as relative humidity, air temperature, and wind speed. The estimation of ETo was performed for arid-warm and warm-temperate climates. In this case, commonly measurable climatic parameters (relative humidity, air temperature, and wind speed) are used, disregarding solar radiation. For the development and validation of our model, we used databases obtained from CIMIS and a database of the El Porvenir station in the state of Coahuila, located in north-central Mexico. In our model performance evaluation, we utilize statistical indicators such as the root mean square error (RMSE) and the coefficient of determination R^2^, comparing the obtained results with the Hargreaves–Samani models and the reference model.

The implementation of our ETo model based solely on temperature, relative humidity, and wind speed data will offer significant benefits to the community. By simplifying the process and reducing dependence on complex meteorological data, this approach becomes more accessible and economically viable, especially valuable in areas with limited resources or less developed meteorological infrastructure. The widespread application of the model, adaptable to at least two different climates (warm temperate, warm arid), will enable efficient irrigation management in agriculture. This will result in water use optimization, significantly contributing to environmental sustainability. The positive impact of this approach will be notably reflected in agricultural planning and water resource management, generating tangible benefits for local communities in terms of sustainability and efficiency. The successful implementation of our model will not only improve water efficiency in agriculture but also result in lower production costs, thus strengthening the economic viability of agricultural communities.

The main contributions of this research are the following:

- The generation of an analytical model using GP, climatic parameters (relative humidity, air temperature, and wind velocity) and the use of expert knowledge to estimate the daily ETo.
- A generalized model to be used in at least two types of climate (arid and temperate) with results that are comparable to the state of the art.
- A methodology that could be employed to discover a function that estimate the ETo under specific restrictions

The article is organized as follows: “Materials and methods” section shows the materials and methods; “Results” section. In “Discussion” section presents the results, “Discussion” section presents the discussion, and “Conclusions and future work” section presents the conclusions and future work.

## Materials and methods

**Figure 1:**
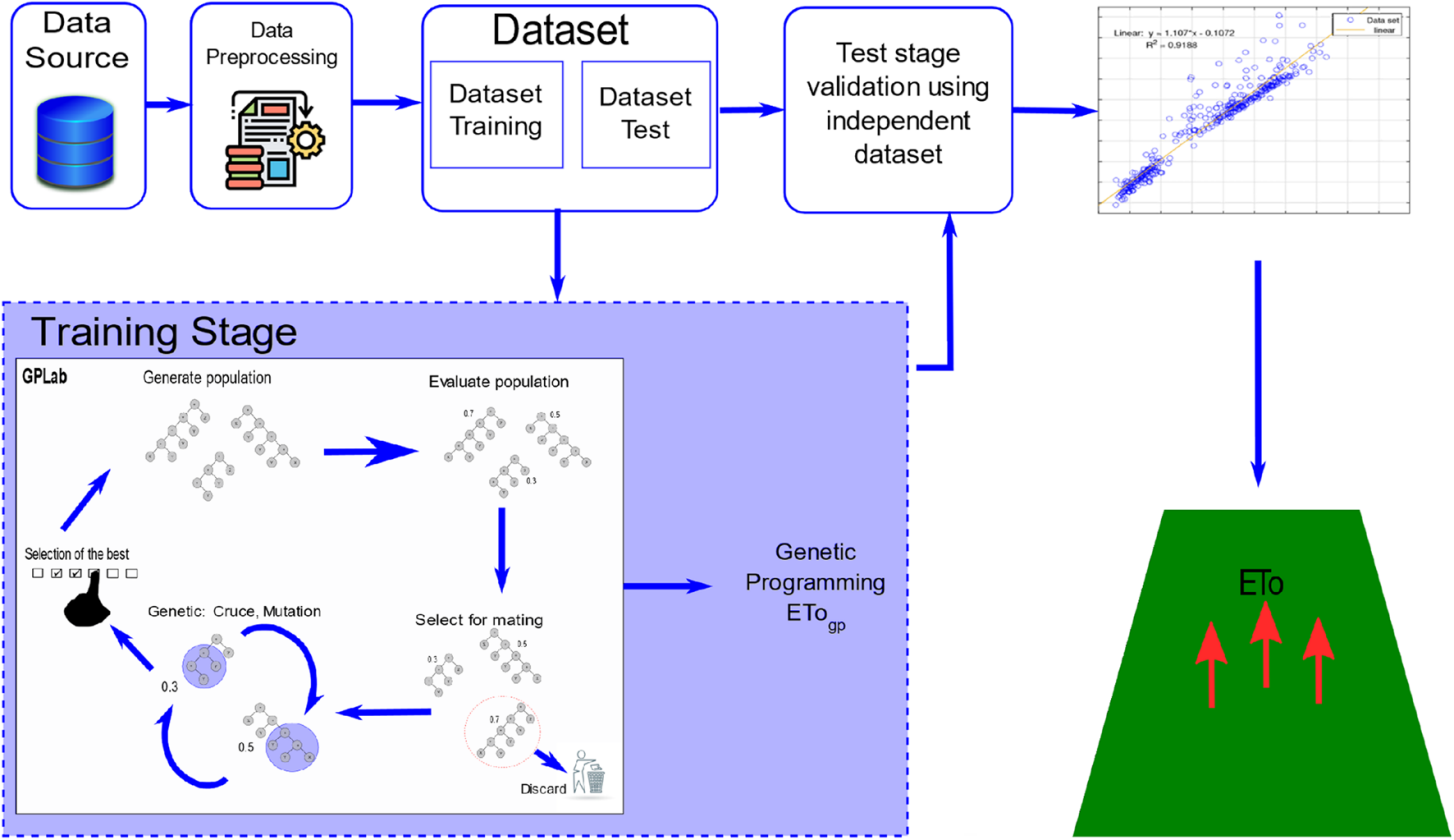
Flowchart of the evolutionary process. From a training dataset, the GP proposes various types of models with the aim of identifying the one that best fits the behavior of ETo within a specific time range. *Source*: Author and collaborators’ work.

In this section, we provide a detailed explanation of the FAO56-PM reference model and the Hargreaves–Samani model, along with some generalities about GP. Subsequently, we elaborate on the model development process, addressing aspects ranging from data acquisition, organization, and preprocessing to the execution of the evolutionary algorithm, as illustrated in Fig. 1. This comprehensive approach aims to offer a complete and understandable view of the methodological framework used in constructing our model, highlighting key stages and the sequence of actions that led to its final formulation.

### The FAO56-PM reference method

The FAO Penman‒Monteith method called (FAO56-PM), Eq. (1), is recommended as the standard method for estimating ETo. However, this estimation poses a challenge when the availability of meteorological data is limited. The FAO56-PM method requires parameters for its application, such as the slope of the vapor pressure curve, *∆* [kPa/°C]; the net surface solar radiation, *R*_*n*_ [MJ m^2^ dia^−1^]; the thermal flux density of the soil, *G* [MJ m^2^ dia^−1^]; the psychrometric constant, *γ* [kPa°C^−1^]; the mean air temperature, *T*_*mean*_ [°C]; the speed of the wind, *u*_2_ [M/s]; the saturation vapor pressure, *e*_*s*_ [kPa]; and the actual vapor pressure, *e*_*a*_ [kPa]. These parameters are a function of air temperature, soil temperature, relative humidity, solar radiation, atmospheric pressure, wind speed, etc.^2^.

In this study, the FAO56-PM method Eq. (1) has been used as a reference to evaluate the results of the investigation due to the absence of experimental values for ETo.

1 $${ETo}=\frac{0.408\Delta \left({R}_{n}-G\right)+\gamma \frac{900}{{T}_{mean}+273}{u}_{2}\left({e}_{s}-{e}_{a}\right)}{\Delta +\gamma \left(1+0.34{u}_{2}\right)}$$

### The Hargreaves–Samani method

The FAO currently recommends the Hargreaves–Samani Eq. (2) in cases where only temperature data are available^21^. This is the result of seeking a standardization of the different existing empirical methods to estimate ETo with reduced data^22–24^.

2 $${ETo}=0.0023{R}_{a}\sqrt{{T}_{max}-{T}_{min}}*\left({T}_{mean}+17.8\right)$$

where *T*_*max*_ represents the maximum temperature [°C]; *T*_*min*_ represents the minimum temperature [°C]; and *R*_*a*_ represents the extraterrestrial radiation [MJ m^2^dia^−1^]. However, the Hargreaves–Samani equation underestimates the ETo value^2^.

### Genetic programming

**Figure 2:**
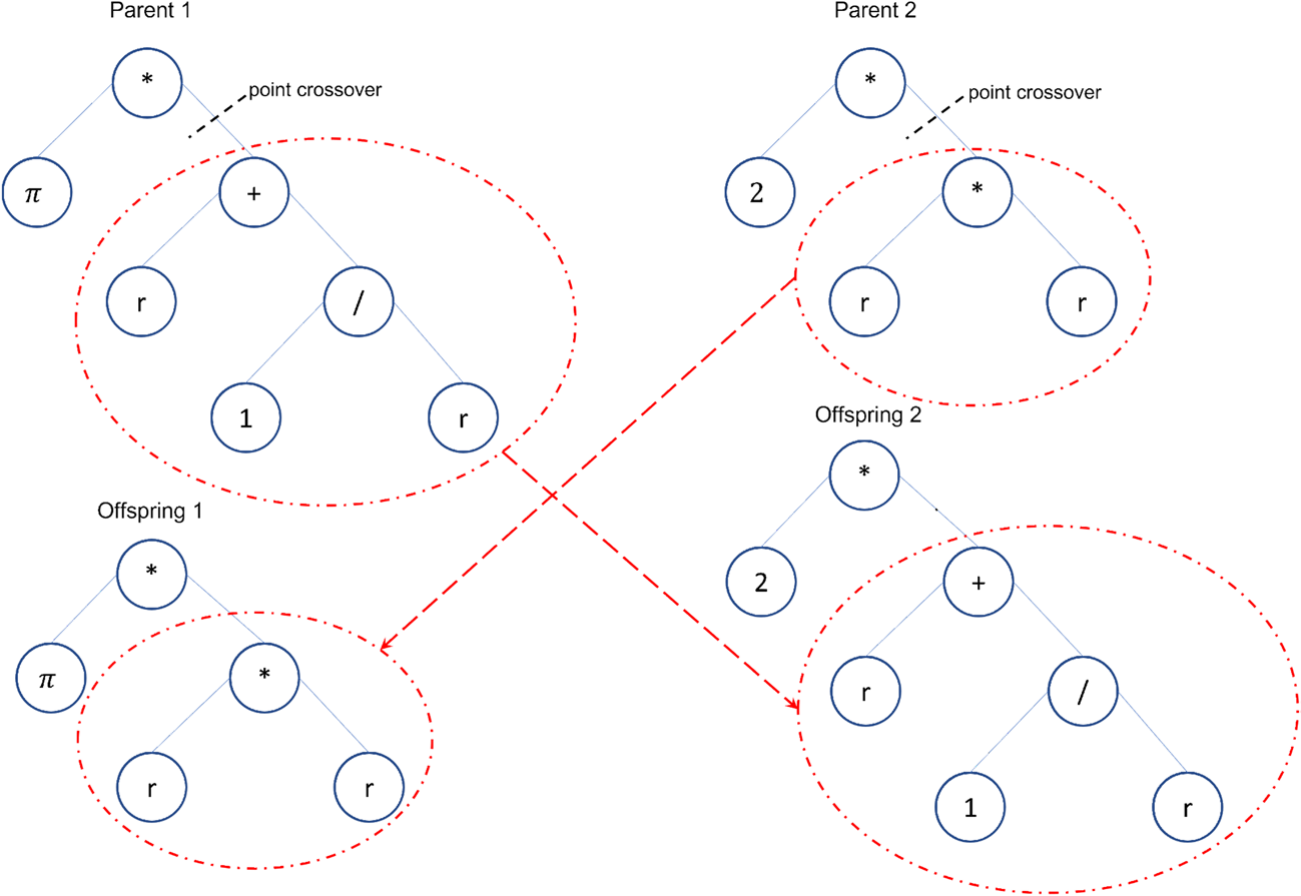
Crossover operator. *Source*: Author and collaborators’ work.

**Figure 3:**
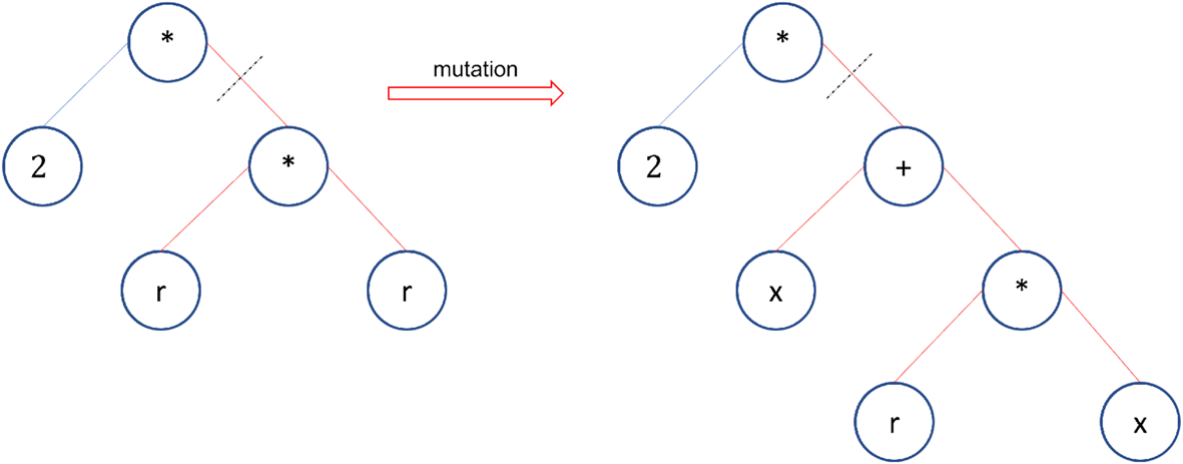
Mutation operator. *Source*: Author and collaborators’ work.

**Figure 4:**
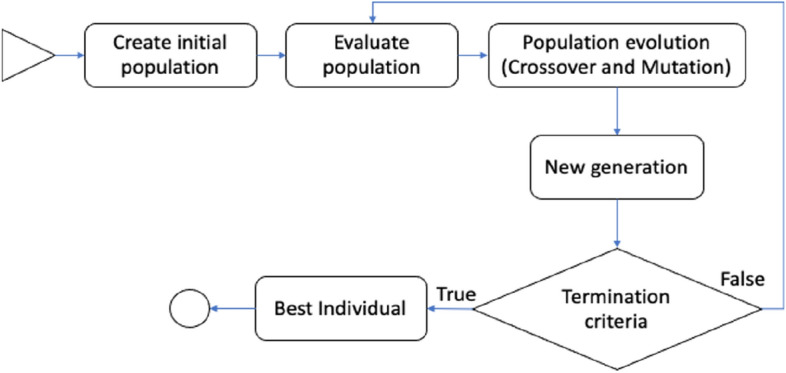
Genetic programming flowchart. *Source*: Author and collaborators’ work.

**Figure 5:**
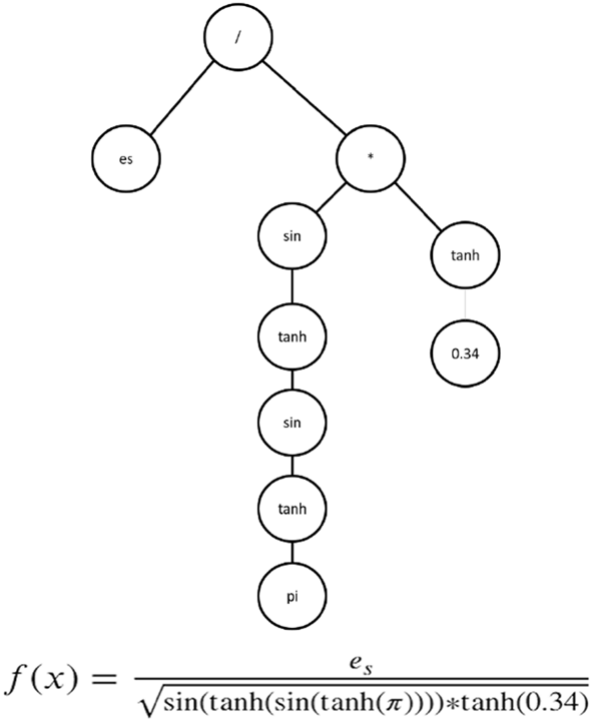
Syntax tree solution. *Source*: Author and collaborators’ work.

Genetic programming (GP) is a domain-independent technique in which computer programs evolve to solve problems. GP is based on the Darwinian principle of survival of the fittest as well as analogies to natural genetic operations such as reproduction and mutation^25^. GP builds a population of individuals (mathematical models) from different combinations of a set of mathematical expressions randomly. Once the population is initialized, the fitness of each individual is evaluated on the basis of some objective function. Fitness is a numerical value that is assigned to each individual based on its performance. The better the fitness of an individual is, the higher the probability of passing to the next generation or having offspring. In each generation, the models are modified by applying genetic operators: selection, crossover, and mutation^25^. Each individual is represented in the form of a syntactic tree. The crossover operator generates new models so that the search space of the problem is thoroughly sampled. The crossover is performed by selecting two parents from the population and exchanging the corresponding subtree structures in a corresponding random area through a randomly chosen point. The crossover operator produces two offspring with different characteristics. The crossover point between Parents 1 and 2 is shown in Fig. 2 with a dotted line; the structures of the corresponding subtrees are swapped, giving rise to Children 1 and 2. The number of models that intersect depends on the probability set in the parameters of the evolutionary algorithm. Mutation involves the random alteration of the syntax tree at the branch or node level. This alteration is made based on the established mutation probability. The mutation introduces new offspring into the population and thus avoids falling into a local optimum. Figure 3 shows the mutation operator. These new models form the basis for the next generation. Each model of the population can be considered to be a potential solution to the problem. Figure 4 shows the flowchart of GP. In GP the coding of individuals is performed in the form of a tree, where the interior nodes represent the functions and the terminal nodes represent the variables and constants (See Fig. 5).

The GP technique has the ability to work with limited data Pioneering research, such as that conducted by^26,27^, has underscored the intrinsic ability of GP to adapt and evolve solutions in environments with small datasets. Specific strategies for handling limited data, including genetic operators and diversity control techniques, have been proposed to enhance the effectiveness of GP in extracting patterns under these conditions. Furthermore, studies such as^28^ have explored GP’s adaptability to problem complexity, enabling the effective representation of patterns even in situations with sparse data.

### Genetic programming to estimate reference evapotranspiration

#### Database

The direct measurement of ETo is complex and is usually estimated indirectly through measurements of climatic parameters.

The databases used in the development of our model were obtained from CIMIS, which houses various weather stations located in different regions of the state of California, United States. A noteworthy feature of CIMIS is that it provides the necessary variables to calculate ETo using the FAO-PM reference model. Additionally, it is important to highlight that CIMIS is an open database, facilitating access and availability of information for the scientific and academic community and one from the state of Coahuila in north-central Mexico. Information on the CIMIS database can be acquired free of charge, from www.cimis.water.ca.gov. Data are available in the database at hourly and daily time scales, and includes solar radiation, air temperature, soil temperature, relative humidity, vapor pressure, wind speed, wind direction and precipitation. The total incoming solar radiation is measured by employing pyranometers at a height of 2.0 m above the ground. Air temperature is measured at a height of 1.5 m above the ground by using a thermistor. Soil temperature is measured at 15 cm (6 inches) below the soil surface. The relative humidity sensor is sheltered in the same enclosure with the air temperature sensor at 1.5 m above the ground. Wind speed is measured by utilizing three-cup anemometers at 2.0 m above the ground. Wind direction is measured by using a wind vane at 2.0 m above the ground. Wind direction values range from 0 to 3608 (both being true north) and are adjusted for declination of the Earth’s axis. Rainfall is measured by employing tipping bucket rain gauges.

The databases for the construction of the model cover a period from 2011 to 2015 and 2019 on a daily scale. The selection of these periods is due to having fewer missing data.

GP, as an evolutionary computational approach, has a remarkable ability to work efficiently with small datasets compared to artificial neural networks, which require a large amount of data. In line with this characteristic, we have decided to use small datasets from CIMIS. This choice does not imply a restriction derived from the availability of the database, as we acknowledge that it is accessible. Instead, it is based on the inherent suitability of GP to extract patterns and generate effective solutions in data-constrained environments. This approach allows us to explore and leverage the capability of GP to address complex problems and extract meaningful knowledge even when the amount of available data is limited. Since an analytical model is obtained, it can be easily verified across different time intervals. In this context, the main objective of the work is to present the methodology for estimating an ETo model. The type of climate was defined based on the climatic classification of^29^. To choose the training and testing databases, geographical diversity and climatic variability were considered as criteria. The databases used for training and testing consist of 4 weather stations located in different geographic locations, with 2 stations having arid-warm climate and the other two with warm-temperate climate, as detailed in Table 2. As for the selection of databases for validation, different stations were chosen compared to those used in the training and testing phases but with similar climates. To validate the model, 4 different weather stations were used, with 3 of them having warm-temperate climate and 1 with arid-warm climate, as shown in Table 3. The attributes of the databases used are: maximum temperature (T_max_ [°C]), minimum temperature (T_min_ [°C]), maximum relative humidity (HR_max_ [%]), minimum relative humidity (HR_min_ [%]), and daily mean wind speed (u_2_ [m/s]).

**Table 2:** Features of the databases used for training and testing.

No	Database	Latitude	Longitude	Elevation (masl)	Climate	Mean Annual temperature (°C)	Mean Annual rainfall (mm)	Period
1	El Porvenir	25.7811	− 103.3130	1112.00	Arid-warm (BWh)	18.0–22.0	250	Feb-Dic 2019
2	Davis	38.5357	− 121.7763	18.288	Warm-temperate (Csa)	16.8	613	2011–15
3	Calipatria	33.0431	− 115.4158	− 33.528	Arid-warm (BWh)	6.0–42.0	76	2011–15
4	McArthur	41.0637	− 121.4560	1008.888	Warm-temperate (Csb)	9.4	1441	2011–15

**Table 3:** Features of the databases used for validation.

No.	Database	Latitude	Longitude	Elevation (masl)	Climate	Mean Annual temperature (°C)	Mean Annual rainfall (mm)	Period
1	Modesto	37.6452	− 121.1877	10.668	warm-temperate (Csa)	17.6	474	2019
2	Oakville	38.4284	− 122.4102	60.655	warm-temperate (Csc)	15.0	594	2015
3	Meloland	32.8061	− 115.4462	− 16.764	arid-warm (BWh)	6.0–42.0	76	2019
4	Ferndale	40.6044	− 124.2431	6.4008	warm-temperate (Csb)	9.9	1617	2019

**Figure 6:**
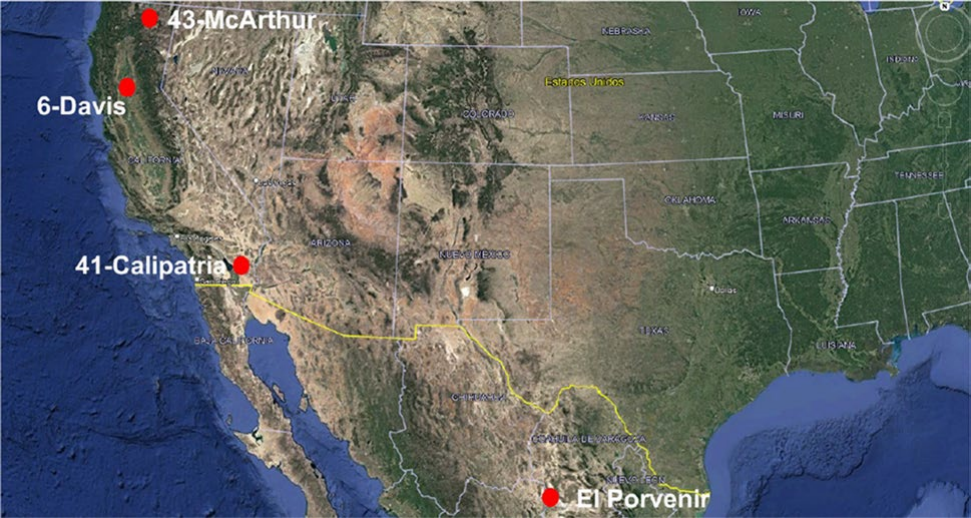
Weather stations used for training and testing.

**Figure 7:**
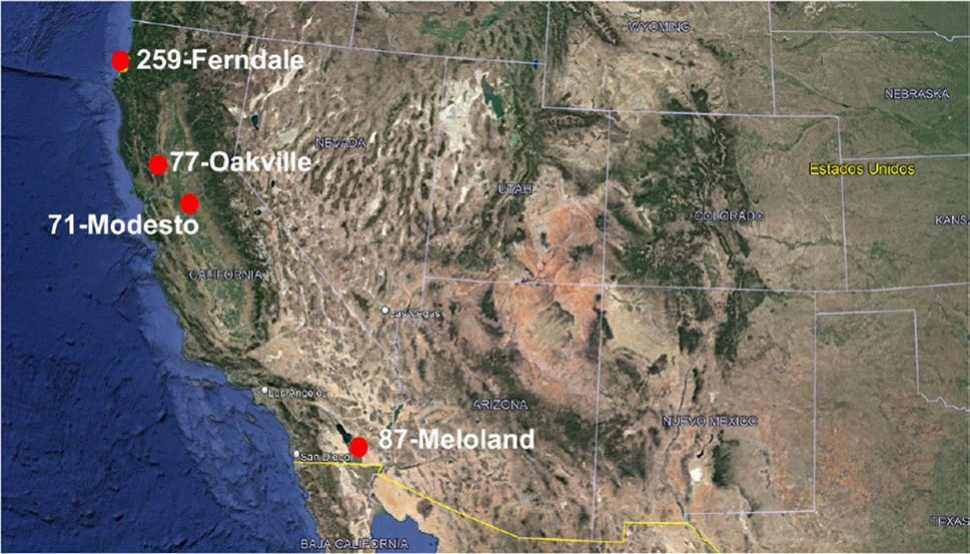
Weather stations used for validation.

Figures 6 and 7 were extracted from Google Maps^30^ and subsequently customized following the guidelines published in^31^. The figures display the geographical locations of the weather stations used in the training, testing, and validation phases of our model.

#### Data preprocessing

An exploratory analysis of the databases was conducted to identify potential incorrect, missing, or improperly recorded data. During this analysis, it was observed that the CIMIS databases contained records with missing data In accordance with^32^, a strategy to address this situation is to use the standard method for imputing missing data, as proposed by^33^. This approach involves replacing missing values with the average under similar meteorological conditions within a time window of ± 7 days.

As a result, 334 records were collected from the El Porvenir station during the period from February to December 2019. Additionally, 1826 records were obtained from each of the CIMIS stations, spanning from January 2011 to December 2015, as shown in Table 2.

Subsequently, a preprocessing operation was carried out to standardize the units in which the original data were registered: the temperature was recorded in [°F] and covered to [°C]; the wind speed data were recorded in [mph] and then converted to [m/s]; and solar radiation was initially recorded in [Ly/day] and then converted to [W/m^2^] using the conversion factor Ly/day/2.065 = [W/m^2^] provided by CIMIS.

The databases from El Porvenir, Davis, Calipatria, and McArthur were used to create three different datasets (DS01, DS02, and DS03), as shown in Table 4. These datasets were utilized in both the training and testing phases.

**Table 4:** Dataset created for model development.

No	Dataset	Contains data from the station	Records
1	DS01	El Porvenir	334
2	DS02	Davis	1826
3	DS03	El Porvenir, Davis, Calipatria, McArthur	5812

The datasets were consolidated as follows: DS01 incorporates data from the El Porvenir station, representing an arid-warm climate (BWh); DS02 includes data from the Davis station with a warm-temperate climate (Csa); and DS03 is formed by integrating data from four meteorological stations (El Porvenir, Davis, Calipatria y McArthur) covering at least two different climates [arid-warm (BWh) and warm-temperate (Csa)]. Table 4 shows the three datasets and their characteristics.

Each daily dataset shown in Table 4 was divided into 80% for training and 20% for testing. Uniform sampling was performed to obtain the test data (i.e., one out of every five data items was taken for the test set, and the remainder was used for the training set). The structure of the daily training and test datasets is shown in Table 5.

**Table 5:** Structure of training and test datasets.

T_max_ (°C)	T_min_ (°)C	HR_max_ (%)	HR_min_ (%)	Daily mean (U_2_) (m s^−1^)	DOY (Day of the Year)
24.9	3.9	69	22	0.55	32
25.4	6.4	76	21	0.70	33
27.8	6.8	69	21	0.59	34
27.4	9.4	66	28	0.56	35
24.8	9.0	73	26	0.74	36
26.7	15.2	60	27	1.3	38

To validate the results, four meteorological stations with different geographical locations than those used in the training and testing phases were included, as detailed in Table 3. These stations were used to create four distinct datasets: DS04, associated with the Modesto station; DS05, linked to the Oakville station; DS06, corresponding to the Meloland station; and DS07, related to the Ferndale station. Table 6 presents the characteristics of the datasets used in the validation process. This approach ensures the robustness of the model by assessing its performance under varied geographical conditions.

**Table 6:** Dataset created to validate the *ETo11* model.

No.	Dataset	Contains data from the station:	Year	Records
1	DS04	Modesto	2019	265
2	DS05	Oakville	2015	342
3	DS06	Meloland	2019	336
4	DS07	Ferndale	2019	149

The choice of the ETo11 model was based on the simplicity of its structure, the number of input parameters, and the accuracy it demonstrated during the training and testing stages, as observed in Table 12. While there are other models available, due to space constraints, we exclusively utilized the ETo11 Model.

#### Construction of the proposed model with genetic programming

For the development of the model, the GPLab library was used in the MatLab environment. This tool proved to be essential for designing and analyzing our model, providing advanced functionalities and facilitating the implementation of specific algorithms needed for our research objectives. The choice of the GPLab library was based on its versatility, effectiveness, and ability to address the particular challenges of our project, enabling us to achieve accurate and meaningful results within the scope of our study.

GP has provided a new way of analyzing and optimizing water resources due to its ability to solve complex problems with one or more objectives. These problems were classified as intractable with traditional methods since their models can be discontinuous and nondifferential, with mixed and integral variables and with a high dimensionality^34^.

Before applying GP to a problem, five preparatory steps must be performed according to John^25^. These five steps consist of determining the set of terminals, the set of functions, the fitness function, the parameters to control the execution, and the method to designate a result.

##### Determining the set of terminals

The set of terminals represents the independent variables of the model not yet discovered. The set of terminals (along with the set of functions) are the elements from which GP attemps to build a model to solve the problem posed. In our research, during the process of selecting the set of functions and terminals, we conducted various experiments using genetic programming. This methodology enables the evolutionary algorithm to discriminate functions and terminals that are not relevant in model construction, preserving only those elements that have a higher degree of utility. Additionally, we relied on the valuable expert knowledge provided by the Penman–Monteith reference model. Since evaluating the Penman–Monteith model involves a series of essential steps, we based our procedures on these, integrating them into the evolutionary algorithm to enhance the robustness and precision of our approach. This combined approach of genetic programming and expert knowledge from Penman–Monteith has proven to be integral in selecting functions and terminals, improving the quality and effectiveness of our models. Table 7 shows the set of terminals chosen for the evolutionary algorithm.

**Table 7:** Terminals used in the evolutionary process.

Index	Terminal	Description	Mathematical expression
1	∆	Slope of the vapor pressure curve	$\Delta =\frac{4098\left[0.618*exp\left(\frac{17.27*T}{T+237.3}\right)\right]}{{\left(T+237.3\right)}^{2}}$
2	*T*_*Max*_	Maximum temperature	
3	*T*_*Min*_	Minimum temperature	
4	*Tprom*	Average temperature	
5	*HR*_*Max*_	Maximum relative humidity	
6	*HR*_*Min*_	Minimum relative humidity	
7	*u*_2_	Wind speed	
8	*rs*	Solar radiation	
9	*d*_*s*_	solar declination	$\delta =0.409*sin\left(\frac{2\pi }{365}|J-1.39\right)$
10	*G*	Soil temperature	
11	*Pi*	pi number	$\pi$
12	*reflexion*	Albedo or reflection coefficient of the crop = 0.23	
13	*gt*	Total degrees	
14	*lt*	Latitude	
15	*P*	Atmospheric pressure	$P={101.3\left(\frac{293-0.0065z}{293}\right)}^{5.26}$
16	*γ*	Const. psychrometric	$\gamma =\frac{{c}_{p}P}{\epsilon \lambda }$
17	*RadSolar*	Solar radiation	
18	*eoT*_*min*_	Saturation vapor pressure temp. min	${e}^{o}{T}_{min}=0.618*exp\left(\frac{17.27*{T}_{min}}{{T}_{min}+237.3}\right)$
19	*eoT*_*max*_	Saturation vapor pressure temp. max	${e}^{o}{T}_{max}=0.618*exp\left(\frac{17.27*{T}_{max}}{{T}_{max}+237.3}\right)$
20	*e*_*s*_	Saturation vapor pressure	${e}_{s}=\frac{{e}^{o}{T}_{min}+{e}^{o}{T}_{max}}{2}$
21	*ea*	Current vapor pressure	${e}_{a}=\frac{{e}^{o}\frac{{T}_{min}*{HR}_{max}}{100}+{e}^{o}\frac{{T}_{max}*{HR}_{min}}{100}}{2}$
22	*es-ea*		
23	*d*_*r*_	Relative Distance from the Earth to the Sun	${d}_{r}=1+0.033*cos\left(\frac{2\pi }{365}|J\right)$
24	*ds*		
25	*w*_*s*_	Radiation Angle	${w}_{s}=arccos\left[-tan\left(\varnothing \right)tan\left(\delta \right)\right]$
26	*N*	Photo period	$N=\frac{24}{\pi }*ws$
27	*R*_*a*_	Extraterrestrial Radiation	${R}_{a}=\frac{24*60}{\pi }{G}_{sc}{d}_{r}$
28	*rs*	Solar radiation	
29	*rns*	net shortwave radiation	
30	*rnl*	net longwave radiation	
31	*rn*	net radiation	
32	*Constant*	0.408	
33	*Constant*	900	
34	*Constant*	273	
35	*Constant*	1	
36	*Constant*	0.34	

##### Determining the set of functions

Functions are the mathematical operators that are applied to the different terminals. In our research, the basic operators +, −, * and / were used in conjunction with √*x*, √*x*, *x*^2^, *x*^3^, *ln*(*x*), exp(*x*), sin(*x*), cos(*x*), and arctan(*x*) functions used in hydrological studies^35,36^. The set of functions included the hyperbolic functions normally used to discover physical phenomena^37^. The set of functions is presented in Table 8.

**Table 8:** Functions used in the evolutionary process.

Index	Function	Expression	Index	Function	Expression
1	Sine	*sin*(*x*)	9	Hyperbolic Tangent	*tanh*(*x*)
2	Cosine	*cos*(*x*)	10	Square Root	$\sqrt x$
3	Tangent	*tan*(*x*)	11	Power	*X*^*y*^
4	Arc Sine	*asin*(*x*)	12	Exponent	$e^{x}$
5	Arc Cosine	*acos*(*x*)	13	Sum	+
6	Arc Tangent	*atan*(*x*)	14	Subtraction	−
7	Hyperbolic Sine	*sinh*(*x*)	15	Multiplication	∗
8	Hyperbolic Cosine	*cosh*(*x*)	16	Division	/

##### Determining the fitness functions

The evolutionary process is governed by the measure of fitness. Each individual in the population is run and then evaluated using the fitness function to determine its performance. The aptitude function (along with the functions and terminals) establishes the search space and allows the quality of the individuals to be evaluated. In this case, it must take positive real values. In our investigation, we chose to apply the RMSE Eq. (3) Use a fitness function to evaluate the difference between the predicted value and the target value. Therefore, the fitness function is defined as follows:

3 $$RMSE=\sqrt{\frac{{\sum }_{i=1}^{n}{\left({P}_{i}-{O}_{i}\right)}^{2}}{n}}$$

where *n* is the total number of data points and *P*_*i*_ and *O*_*i*_ are the predicted and target ETo values, respectively. The better the fitness of an individual is, the higher the probability of passing to the next generation.

##### Determining the parameters of the evolutionary algorithm

The main parameters for controlling the execution of the evolutionary algorithm are the population size, the maximum number of generations in the initialization method, the selection method, the crossover probability, and the mutation probability. In our research, the values assigned to the parameters are observed in Table 9.

**Table 9:** Parameters of the evolutionary algorithm.

Parameter	Value
Population size	200
Maximum number of generations	200
Initialization method	Ramped half-and-half
Selection method	‘lexictour’
Crossover probability	80%
Mutation probability	20%

##### Determining the method to designate a result

Each execution requires the specification of a termination criterion to decide when to terminate and a method of designating results. The solutions were obtained by taking an RMSE < 1 in training. To stop the evolutionary algorithm, we use the maximum number of generations established in the algorithm parameters as the stop criterion.

In our research, three datasets from different climates based on^29^ classification were tested as input for the formulation of models with GP see Table 4. After exploratory testing, it was necessary to adjust the configuration of the process. The evolutionary algorithm was run fifty times for the DS01 and DS02 datasets, obtaining 2076 and 1408 solutions, respectively. For the DS03 dataset, the evolutionary algorithm was executed 30 times using the same parameters established, resulting in 270 solutions. In total, the evolutionary process obtained 3754 solutions (see Table 10).

**Table 10:** Solutions found.

Models	
Dataset	Solutions
DS01	2076
DS02	1408
DS03	270
Total	3754

The evaluation of the models was carried out with 20% of the data reserved for testing of the DS01, DS02, and DS03 datasets. The evaluation was carried out by calculating the RMSE and the coefficient of determination R^2^; this last measure was not considered as part of the optimization of the model. RMSE and R^2^ are two metrics commonly used in the context of predictions and regression models to assess the performance of a model compared to actual values.

The RMSE and R^2^ metrics are commonly used in forecasting to assess both the accuracy and the quality of fit of the model. RMSE provides a measure of how close the model’s predictions are to the actual values, while R^2^ provides information about the proportion of variability that the model is capturing compared to the total variability in the data. Together, these metrics offer a comprehensive view of the model’s performance in terms of accuracy and ability to explain variability in the data. RMSE and R^2^ are calculated using Eqs. (3) and (4), respectively.

4 $${R}^{2}=\frac{{\left[{\sum }_{i=1}^{n}\left({O}_{i}-{\overline{O}}_{i}\right)\left({P}_{i}-{\overline{P}}_{i}\right)\right]}^{2}}{{\sum }_{i=1}^{n}\left({O}_{i}-\overline{{O}_{i}}\right){\sum }_{i=1}^{n}\left({P}_{i}-\overline{{P}_{i}}\right)}$$

where *n* is the total number of data points, and *P*_*i*_ and *O*_*i*_ are the predicted *ETo* and, and ${\overline{O}}_{i}$ and ${\overline{P}}_{i}$ target *ETo* values, respectively.

### Ethical approval and consent to participate

In the course of our investigation, no experimental procedures involving human subjects were undertaken.

## Results

### Evolution Statistics

**Figure 8:**
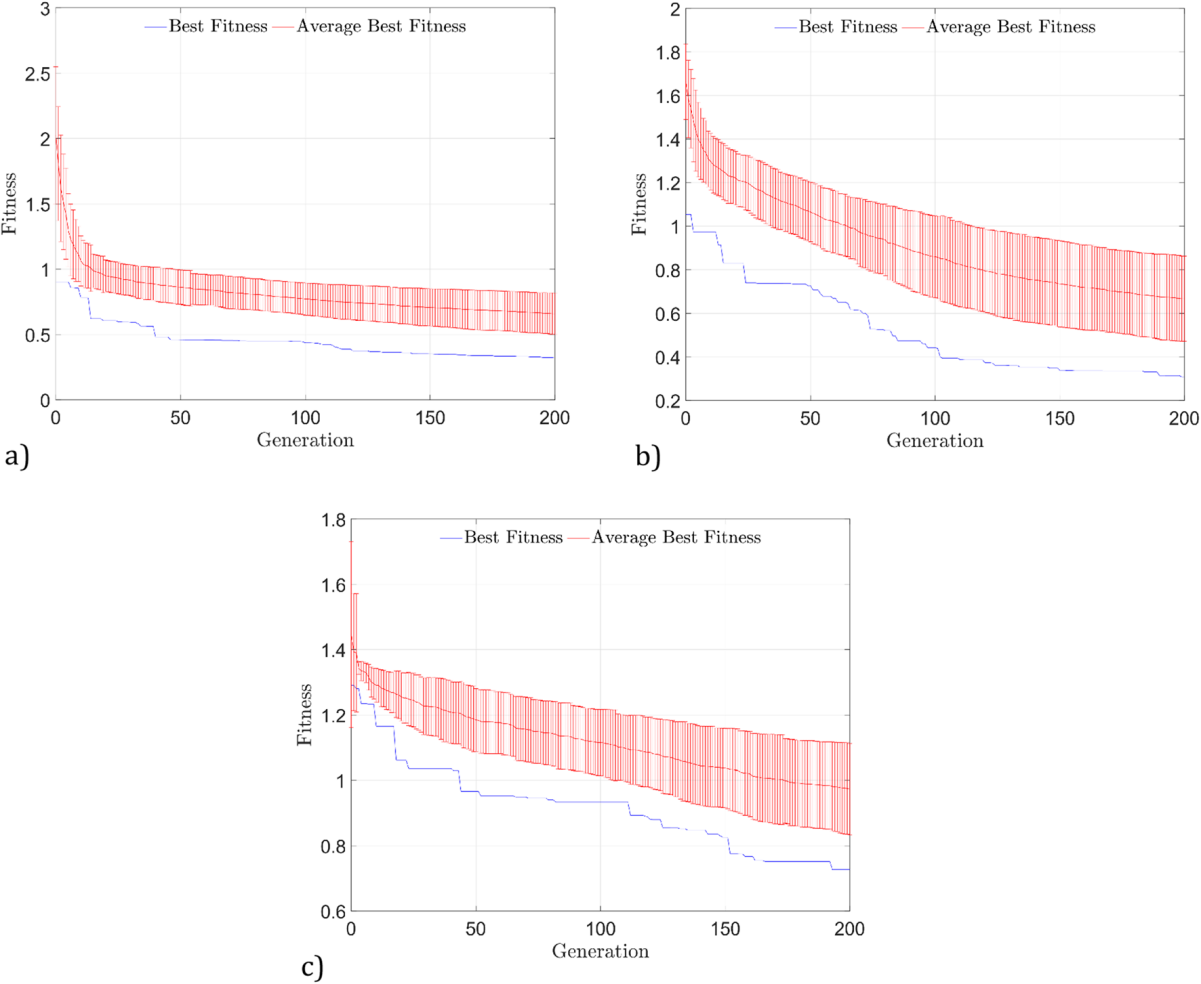
Best fit, the average of the best fits, and the standard deviation of the 50 runs for the DS01 and DS02 datasets and the 30 runs for the DS03 dataset.

This section presents a statistical analysis of the evolutionary process for the DS01, DS02, and DS03 datasets. The objective is to show the convergence rate of the proposed algorithm. Figure 8 shows the best fit, the average of the best fits, and the standard deviation of the 50 runs for the DS01 and DS02 datasets and the 30 runs for the DS03 dataset.

Note that, on average, solutions with a fitness measure less than one were found in the 15th, 65th, and 174th generations. The minimum fitness was reached for the three datasets around the 200th generation, and the average fitness converged to 0.9036, 0.9734 and 0.9668 from the 1st, 3st and 44th generations for the DS01, DS02 and DS03 datasets, respectively.

### Experimental results

The number of solutions found with GP was 3754, from which the fourteen best solutions were selected. Priority was given to those with the best aptitude, an RMSE less than one, the least structural complexity, and the lowest number of input parameters. The selected models are shown in Table 11. The models were labeled EToi (where i represents a consecutive number).

**Table 11:** Models obtained with genetic programming.

Model	Mathematical expression	RMSE	Data set
*ETo1*	$ETo=\frac{{e}_{s}}{0.4718}$	1.041	DS01
*ETo2*	$ETo=arctan\left(\frac{{u}_{2}}{{d}_{r}+{e}_{s}+\frac{{e}_{s}}{{d}_{r}}}\right)+\frac{\frac{{e}_{s}}{{d}_{r}}}{{d}_{r}}+\frac{\frac{\frac{{e}_{s}}{{d}_{r}}}{{d}_{r}}}{{d}_{r}}$	0.952	DS01
*ETo3*	$ETo=\sqrt{{e}_{a}+{u}_{2}}+2{d}_{s}acos\left({u}_{2}\sqrt{\sqrt{{e}_{a}+{u}_{2}*{u}_{2}}+\sqrt{{d}_{s}}}\right)+{e}^{o}{T}_{max}$	0.898	DS01
*ETo4*	$ETo={d}_{s}+2{e}_{s}+\frac{{e}_{s}-{e}_{a}}{sinh\left({e}_{s}\right)}$	0.980	DS01
*ETo5*	$ETo={e}^{o}{T}_{max}-\frac{tanh\left({T}_{min}\right)}{{u}_{2}}+{e}^{o}{T}_{min}+{d}_{s}+tanh\left({HR}_{max}\right)$	0.889	DS01
*ETo6*	$ETo=\sqrt{{e}_{s}+{u}_{2}+tanh\left({d}_{s}+{R}_{a}\right)}+2{d}_{s}+{e}_{s}+tanh$)	0.882	DS01
*ETo7*	$ETo={w}_{s}\left(acos\left({lt}^{{u}_{2}}\right)+{d}_{s}+\Delta +{e}_{s}-{e}_{a}\right)$	0.794	DS03
*ETo8*	$$\begin{aligned} ETo & = tanh\left( {\frac{{d_{s} + tanh\left( {tanh\left( {lt} \right)} \right)}}{{1 + tanh\left( {atan\left( { + tanh\left( {e_{s} - e_{a} } \right)} \right) + tanh\left( {2e^{o} T_{max} + tanh\left( {HR_{min} } \right) + tanh\left( {d_{s} } \right)} \right)} \right)}}} \right) + d_{s} \\ & \quad + \frac{{u_{2} + tanh\left( {d_{s} } \right) + d_{s} }}{{atan\left( {atan\left( {\Delta + e^{o} T_{max} } \right) + atan\left( {sin\left( {e^{o} T_{max} } \right)} \right)} \right) + tanh\left( \Delta \right) + tanh}} \\ \end{aligned}$$	0.639	DS02
*ETo9*	$ETo=\sqrt{{u}_{2}+{d}_{s}}+2\left(e|s-{e}_{a}\right)+2{d}_{s}+\sqrt{{d}_{s}}$	0.758	DS02
*ETo10*	$ETo=3{d}_{s}+2\left({e}_{s}-{e}_{a}\right)+atan\left({u}_{2}\right)+atan\left(atan\left(atan\left(atan\left(acos\left(900\right)+sin\left(atan\left({u}_{2}\right)+atan\left(900\right)+sin\left({u}_{2}\right)\right)+{u}_{2}\right)\right)\right)\right)+{u}_{2}$	0.826	DS02
*ETo11*	$ETo=atan\left({u}_{2}\left({e}_{s}-{e}_{a}\right)\right)+{d}_{s}+2atan\left({d}_{s}\right)+atan\left(ds+atan\left({e}_{s}\right)\right)$	0.693	DS02
*ETo12*	$ETo={e}^{o}{T}_{max}+{d}_{s}+2tanh\left({d}_{s}\right)+\frac{{d}_{s}+\left(\frac{{d}_{s}+{u}_{2}}{\pi }\right)+{u}_{2}}{\pi }$	0.889	DS02
*ETo13*	$ETo={d}_{s}+{e}^{o}{T}_{max}*{{u}_{2}}^{reflexion}$	0.950	DS02
*ETo14*	$ETo={e}^{o}{T}_{max}+{{u}_{2}}^{4}*0.1325*3{d}_{s}$	0.872	DS02
*H–S*	${ETo}=0.0023{R}_{a}\sqrt{{T}_{max}-{T}_{min}}*\left({T}_{mean}+17.8\right)$		
*FAO56-PM*	${ETo}=\frac{0.408\Delta \left({R}_{n}-G\right)+\gamma \frac{900}{{T}_{mean}+273}{u}_{2}\left({e}_{s}-{e}_{a}\right)}{\Delta +\gamma \left(1+0.34{u}_{2}\right)}$		

ETo, reference evapotranspiration; H–S, Hargreaves–Samani.

The structure of the *ETo1* and *ETo4* models is a function of temperature; the structure of the *ETo2*, *ETo6*, *ETo7*, *ETo9*, *ETo10*, *ETo12*, *ETo13*, and *ETo14* models is a function of temperature and wind speed; and the structure of the *ETo3*, *ETo5*, *ETo8* and *ETo11* models is a function of relative humidity, temperature, and wind speed.

In this case, simple models such as ETo1 were obtained, which is a function of saturation vapor pressure, but the results outperform the Hargreaves–Samani model in the DS01 dataset, which corresponds to arid-warm climate. Similarly, some models include parameters such as solar declination, which are expressed solely in terms of the day of the year.

#### Evaluation of the models

Table 12 summarizes the performance of the models measured by RMSE and R^2^ for all the datasets shown in Table 4 used to develop the model. It can be observed that the evolutionary approach with GP is capable of learning complex and nonlinear relationships that are difficult to model with conventional techniques. For example, the models have a range of RMSE between 0.639 and 2.054, and R^2^ between 0.521 and 0.925 with 20% of the datasets used for model development.

**Table 12:** Statistical indices where we can observe that the ETo11 model shows greater consistency across the three datasets.

Model	DS01				DS02				DS03			
	Training		Test		Training		Test		Training		Test	
	RMSE	R^2^	RMSE	R^2^	RMSE	R^2^	RMSE	R^2^	RMSE	R^2^	RMSE	R^2^
ETo1	1.131	0.763	1.041	0.775	1.720	0.601	1.706	0.600	1.839	0.679	1.830	0.683
ETo2	0.973	0.823	0.952	0.805	1.663	0.702	1.654	0.700	1.819	0.731	1.812	0.735
ETo3	0.895	0.869	0.898	0.829	1.639	0.811	1.644	0.804	1.657	0.836	1.656	0.841
ETo4	1.094	0.790	0.980	0.797	1.581	0.672	1.575	0.669	1.655	0.736	1.643	0.741
ETo5	0.894	0.849	0.889	0.831	1.861	0.573	2.036	0.521	1.945	0.680	1.959	0.680
ETo6	0.878	0.874	0.882	0.838	1.664	0.815	1.680	0.807	1.590	0.830	1.594	0.833
ETo7	1.723	0.752	1.597	0.723	0.852	0.887	0.847	0.883	0.834	0.892	0.794	0.901
ETo8	1.783	0.794	1.738	0.747	0.688	0.918	0.639	0.925	0.779	0.908	0.761	0.910
ETo9	1.726	0.753	1.632	0.709	0.806	0.887	0.758	0.896	0.898	0.889	0.869	0.894
ETo10	1.793	0.770	1.637	0.746	0.864	0.871	0.826	0.876	0.922	0.883	0.890	0.889
ETo11	1.837	0.809	1.756	0.759	0.753	0.901	0.693	0.910	0.839	0.898	0.807	0.904
ETo12	1.579	0.870	1.538	0.815	0.904	0.871	0.889	0.871	1.021	0.871	1.006	0.874
ETo13	1.804	0.881	1.769	0.812	0.915	0.861	0.950	0.845	1.083	0.829	1.076	0.830
ETo14	2.092	0.860	2.054	0.793	0.923	0.849	0.872	0.860	1.004	0.851	0.996	0.849
H–S	2.501	0.683	2.374	0.631	1.157	0.759	1.177	0.741	1.016	0.834	0.979	0.854

The structural analysis of the models provides interesting insight into the strategies identified by the evolutionary process to combine functions and terminals.

**Figure 9:**
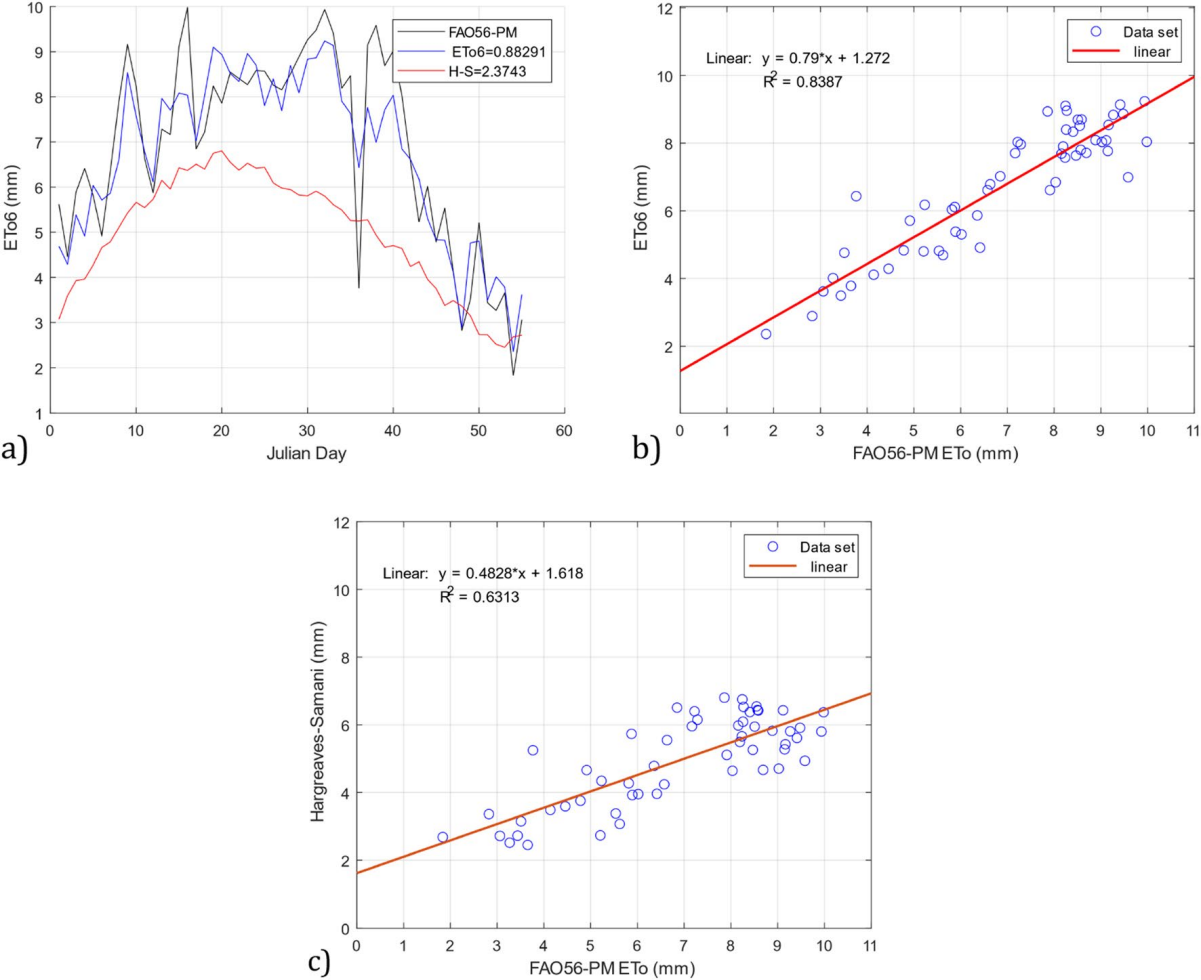
Comparison of the *ETo6* and Hargreaves–Samani models with the FAO56-PM reference model using test data from the DS01 dataset. (**a**) RMSE *ETo6* and Hargreaves–Samani, (**b**) R^2^ *ETo6*, and (**c**) R^2^ Hargreaves–Samani.

In Table 12, the results show that the *ETo6* model with input parameters of temperature and wind speed was the best model with the test data of the DS01 dataset, with RMSE = 0.882 and R^2^ = 0.838. The scatter plots and time series in Fig. 9 show that the *ETo6* model outperformed the Hargreaves–Samani model. For example, in Fig. 9a, the *ETo6* model obtained an RMSE = 0.882, while the Hargreaves–Samani model obtained an RMSE = 2.374. On the other hand, in Fig. 9b, the *ETo6* model obtained R^2^ = 0.8387, and the Hargreaves–Samani model presented in Fig. 9c reached R^2^ = 0.631. This may be because the model was developed using the DS01 dataset.

**Figure 10:**
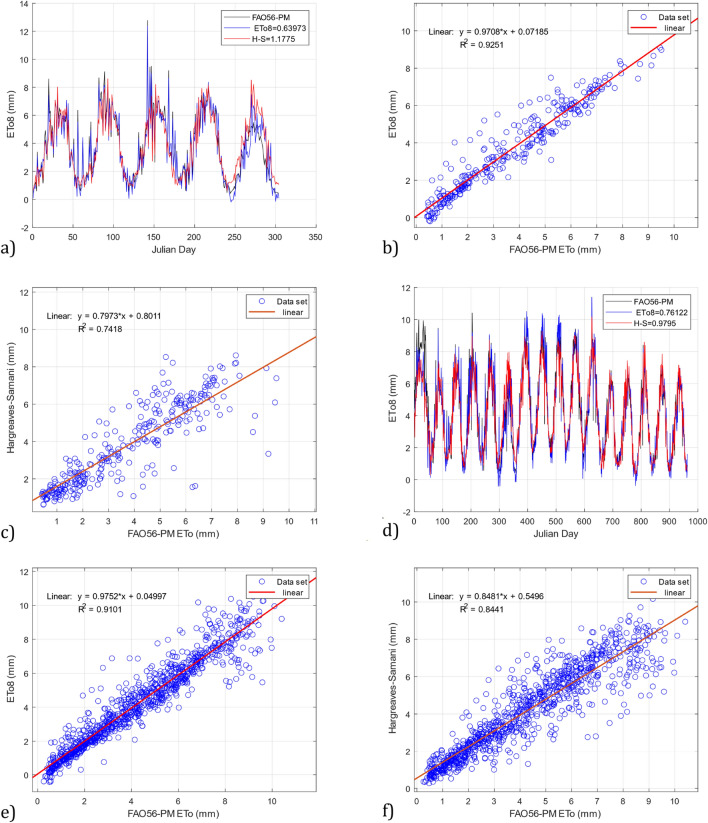
Comparison of the *ETo8* model and the Hargreaves–Samani model with the FAO56-PM model using the datasets DS02 and DS03. (**a**) RMSE *ETo8* and Hargreaves–Samani for DS02, (**b**) R^2^ *ETo8* for DS02, (**c**) R^2^ Hargreaves–Samani for DS02, (**d**) RMSE *ETo8* and Hargreaves–Samani for DS03, (**e**) R^2^ *ETo8* for DS03, and (**f**) R^2^ Hargreaves–Samani for DS03.

The *ETo8* model that uses relative humidity, temperature, and wind speed input parameters obtained more accurate results in estimating the ETo with the DS02 and DS03 datasets than the Hargreaves–Samani model. For the DS02 dataset, Fig. 10a shows a value of RMSE = 0.639 for the *ETo8* model, outperforming the Hargreaves–Samani model, which reached a value of RMSE = 1.177. Figure 10b shows a value of R^2^ = 0.925 for the *ETo8* model, while Fig. 10c shows a value of R^2^ = 0.741 obtained with the Hargreaves–Samani model. Likewise, for the DS03 dataset, Fig. 10d shows an RMSE = 0.761 for the *ETo8* model, while the Hargreaves–Samani model shows an RMSE = 0.979. Figure 10e shows a value of *R*^*2*^ = 0.910 for the *ETo8* model, and Fig. 10f shows a value of R^2^ = 0.844 for the Hargreaves–Samani model.

According to the results presented in Fig. 10, the model developed with GP *ETo8* considerably outperforms the Hargreaves–Samani model. However, the model has a complex structure.

On the other hand, the model *ETo11* Eq. (5) presents a relatively simple structural formulation with a range of results in the evaluation stage with an RMSE between 0.693 and 1.756 and R^2^ between 0.759 and 0.910 for the three datasets. Therefore, even though the *ETo11* model was not the best model found, it was selected for the validation stage. It presents important characteristics to consider since only input parameters that depend on relative humidity, temperature and wind speed and its results outperform the Hargreaves–Samani model on all three datasets used for testing.

#### Model validation

To validate the *ETo11* model, Eq. (5) Developed with GP, the DS04, DS05, DS06, and DS07 datasets were used with arid-warm and warm-temperate climates obtained from locations other than those used for their training. Their characteristics are shown in Table 6. The Hargreaves–Samani model was included to compare the performance between these two models. Table 13 shows the values of *RMSE* and *R*^2^ obtained with the *ETo11* and Hargreaves–Samani models.

**Table 13:** Statistical indices of the *ETo11* and Hargreaves–Samani models.

DataSets	ETo11		Hargreaves–Samani	
	RMSE	R^2^	RMSE	R^2^
DS04	0.314	0.978	0.635	0.916
DS05	0.682	0.918	0.710	0.918
DS06	1.483	0.818	1.616	0.767
DS07	0.292	0.937	0.400	0.867

5 $$ETo11=atan\left({u}_{2}\left({e}_{s}-{e}_{a}\right)\right)+{d}_{s}+2atan\left({d}_{s}\right)+atan\left(ds+atan\left({e}_{s}\right)\right)$$

**Figure 11:**
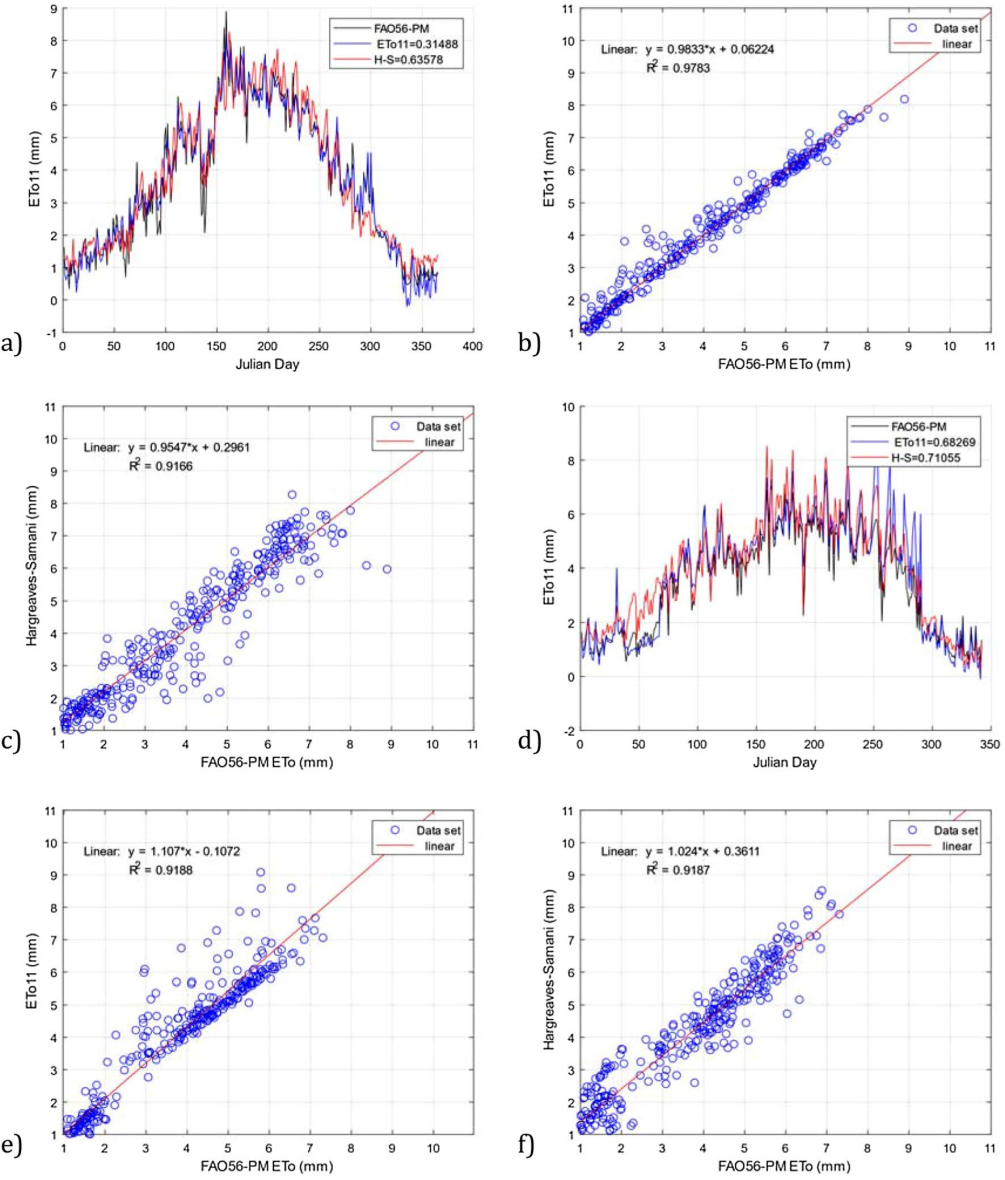
Comparison of the *ETo11* model and the Hargreaves–Samani model with the FAO56-PM model using the datasets DS04 and DS05. (**a**) RMSE *ETo11* and Hargreaves–Samani for DS04, (**b**) R^2^ *ETo11* for DS04, (**c**) R^2^ Hargreaves–Samani for DS04, (**d**) RMSE *ETo11* and Hargreaves–Samani for DS05, (**e**) R^2^ *ETo11* for DS05, and (**f**) R^2^ Hargreaves–Samani for DS05.

**Figure 12:**
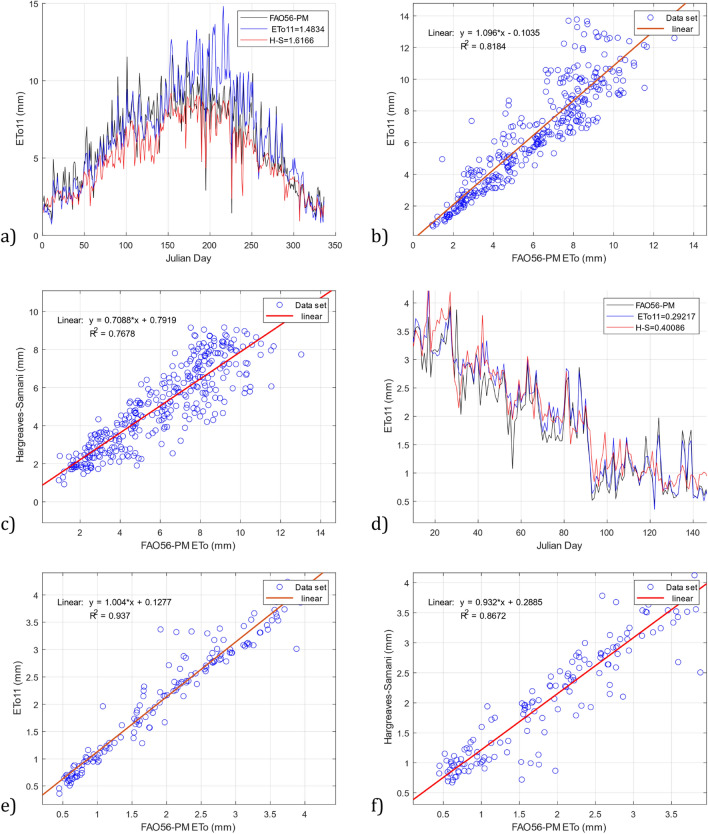
Comparison of the *ETo11* model and the Hargreaves–Samani model with the FAO56-PM model using the datasets DS06 and DS07. (**a**) RMSE *ETo11* and Hargreaves–Samani for DS06, (**b**) R^2^ *ETo11* for DS06, (**c**) R^2^ Hargreaves–Samani for DS06, (**d**) RMSE *ETo11* and Hargreaves–Samani for DS07, (**e**) R^2^ *ETo11* for DS07, and (**f**) R^2^ Hargreaves–Samani for DS07.

The scatter and time series plots in Figs. 11 and 12 show that the *ETo11* model has greater accuracy than the Hargreaves–Samani model with respect to the FAO56-PM model of reference for the four datasets used for validation. For example, Fig. 11a shows greater precision by the *ETo11* model with a value of RMSE = 0.314 than the Hargreaves–Samani model with a value of RMSE = 0.635. Similarly, by means of a linear regression analysis, it can be observed in Fig. 11b,c that our model is a better fit than the Hargreaves–Samani model, with R^2^ =  0.978 against R^2^ = 0.916 with the DS04 dataset.

For the DS05 dataset, the results continue to be favorable for the *ETo11* model, as shown in Fig. 11d. Our model has an RMSE = 0.682 compared to an RMSE = 0.710 from the Hargreaves–Samani model. On the other hand, Fig. 11e,f show the results of the linear regression analysis, where it can be seen that the models obtain similar results in the statistical index R^2^, with a value of 0.918 for the two models. For the DS06 dataset, it can also be observed that the *ETo11* model outperforms the Hargreaves–Samani model. Figure 12a shows values of RMSE = 1.483 obtained with our model compared to the Hargreaves–Samani model with RMSE = 1.616. Figure 12b,c show the results of R^2^ with values of 0.818 and 0.767 for the *ETo11* and Hargreaves–Samani models, respectively, with the *ETo11* model showing greater precision. As a result, our model aims to adhere to the reference data in the different validation datasets, making its behavior similar in Figs. 11 and 12.

These results were obtained for an arid-warm climate. However, to validate our *ETo11* model in a different climate, we used the DS07 dataset with a warm-temperate climate. Similarly, the time series plots in Fig. 12d show that our model outperforms the Hargreaves–Samani model by a lower magnitude with values of RMSE = 0.292 compared to RMSE  = 0.400. The scatter plot of Fig. 12e also shows an index of R^2^ = 0.937 compared to the values of R^2^ = 0.8672 of Fig. 12f, and it becomes clear that our model outperforms the Hargreaves–Samani model.

**Figure 13:**
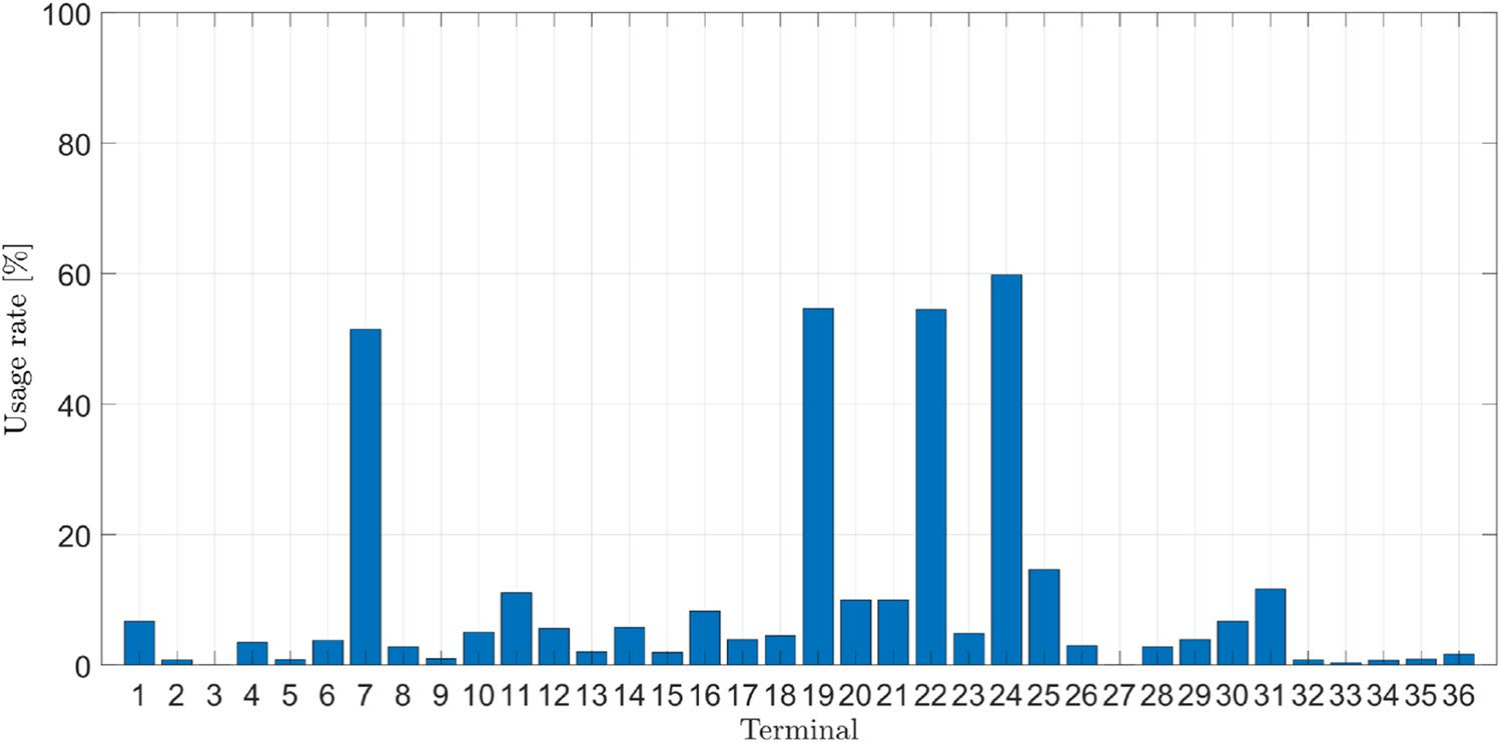
Frequency of occurrence of the terminal set.

**Figure 14:**
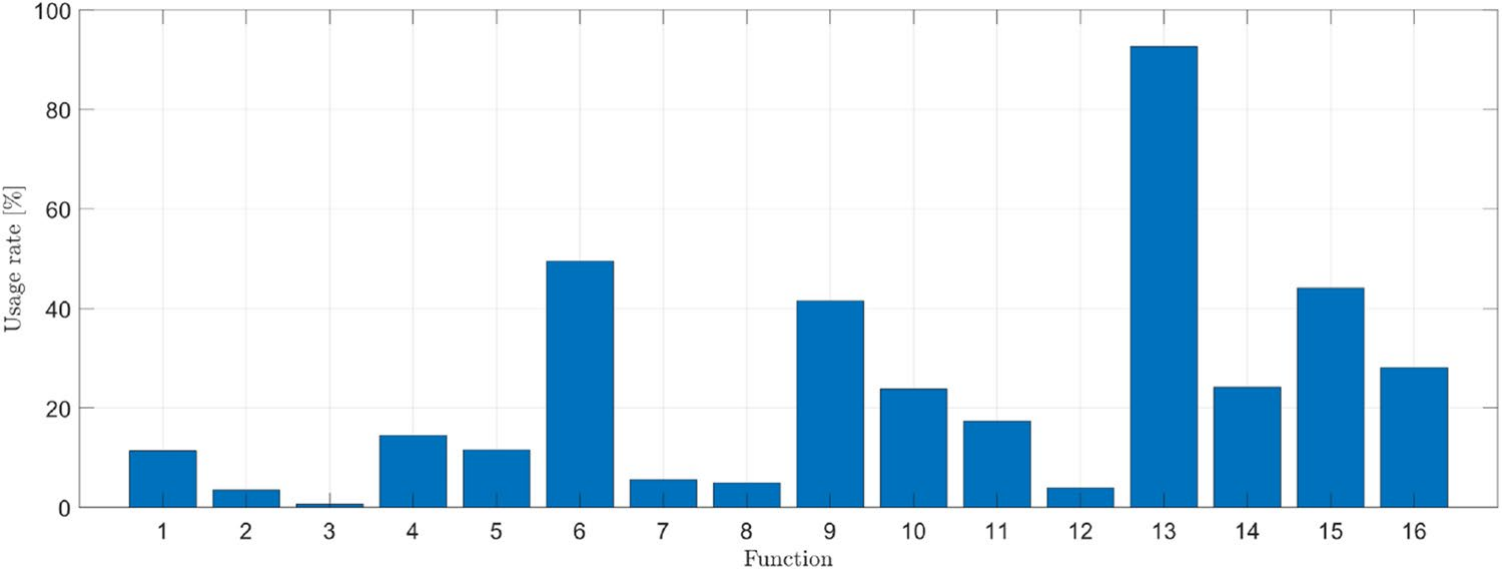
Frequency of occurrence of the function set.

The structural analysis of the ETo models presented in Table 11 offers an intriguing perspective into the strategies identified by the evolutionary process to combine the primitive set (functions and terminals). In this study, we employed a frequency of use unit, representing how often an element is utilized to generate a new model. Figures 13 and 14 depict the occurrence frequency of the function and terminal sets. The function and terminal numbers correspond to those assigned in the description provided in Tables 4 and 5.

Regarding the elements belonging to the terminal set, it can be observed that ws (solar radiation angle at sunset) has the highest occurrence frequency, being used in 59.8% of the generated models. It is followed by e°T_max_ (saturation vapor pressure at max. temp.), which has an occurrence frequency of 54.62%, and finally, the saturation vapor pressure has a frequency of 54.47%. It is noted that the GP process did not utilize the majority of provided terminals, indicating that the GP algorithm identified them as not strictly necessary for estimating ETo. Moreover, it is important to highlight that the presented ETogp models do not directly employ the basic climatic parameters. However, they are implicitly used in variables representing expert knowledge, such as saturation vapor pressure (e_s_), solar declination (d_s_), vapor pressure deficit (e_a_), and vapor pressure at minimum and maximum temperature (e°T_max_, e°T_min_), all of which belong to the Terminal set. For instance, saturation vapor pressure (e_s_) is derived from vapor pressure at minimum temperature (e°T_min_) and vapor pressure at maximum temperature (e°T_max_), both of which are functions of minimum and maximum temperatures. Similarly, the vapor pressure deficit (e_a_) is derived from the vapor pressure at minimum temperature (e°T_min_), vapor pressure at maximum temperature (e°T_max_), minimum relative humidity (HR_min_), and maximum relative humidity (HR_max_). This is intriguing since only temperature and relative humidity climatic parameters are used, suggesting that GP can identify fundamental climatic parameters for estimating ETo.

In the case of the function set, the importance of each operator was more evenly distributed. The addition operator was used in 92.61% of cases, while the sine operator was the least used at 11.36%. This is intriguing as GP can construct structurally simple models for estimating ETo. This finding suggests that the GP algorithm recognized the relationship between fundamental yet effective climatic parameters in estimating the ETo phenomenon.

#### Statistical significance

In order to assess the statistical significance of the data, Student’s t-tests were conducted for each of the datasets used in the validation, as detailed in Table 14.

**Table 14:** Student’s t-test.

Metrics	Dataset DS04		Dataset DS05		Dataset DS06		Dataset DS07	
	Eto11	FAO-PM	Eto11	FAO-PM	Eto11	FAO-PM	Eto11	FAO-PM
Mean	3.6647	3.6637	3.7482	3.4832	6.4708	6.0001	2.0660	1.9304
Variance	4.5185	4.5721	4.4247	3.3184	10.5677	7.2040	1.0696	0.9941
Observations	365	365	342	342	336	336	149	149
Pooled variance	4.5453		3.8715		8.8858		1.0318	
Hypothesized mean difference	0		0		0		0	
Degrees of freedom	728		682		670		296	
t Statistic	0.0057		1.7614		2.0465		1.15237	
P(T ≤ t) One-Tail	0.4976		0.0393		0.0205		0.1250	
Critical t-value (One-Tail)	1.6469		1.6470		1.6471		1.6500	
P(T ≤ t) Two-tail	0.9953		0.0786		0.0410		0.2500	
Critical t-value (Two-Tail)	1.9632		1.9634		1.9635		1.9680	
Significance level	0.05		0.05		0.05		0.05	

The results obtained with a 5% tolerance conclusively indicate that there is no significant difference between the means of the two models, ETo11 and FAO-PM. In this context, it cannot be stated that the models differ significantly in terms of their performance; therefore, both could be considered statistically equivalent based on the data analysis. This finding supports the consistency and reliability of both models in estimating ETo.

## Discussion

In this study, a methodology was presented for developing analytical models of ETo using GP. Unlike genetic algorithms that optimize the parameters of a given model, GP allows for obtaining a new formulation that fits the acquired data. Thus, the advantage of the GP approach lies in its open-box or white-box characteristic. If we were to use a black-box approach (e.g., neural networks, fuzzy logic, and most statistical approaches), we would hardly be able to explicitly uncover the relationship among the climatic variables considered in the ETo phenomenon.

The proposed methodology offers great flexibility, as it can adapt to data from various climate types and account for constraints on these data. Thus, this study aimed to construct an ETo model that only takes into account climatic variables commonly measurable at a meteorological station, such as temperature, relative humidity, and wind speed.

By implementing the proposed methodology, a total of 3754 models were successfully generated, all of which demonstrated a substantial improvement in ETo estimation compared to the Hargreaves–Samani model. However, a meticulous selection process was carried out, choosing only fourteen of these models based on criteria such as their accuracy, input parameters, and structural complexity. During the training phase, a noticeable superiority was evident, reaching up to 27% compared to the Hargreaves–Samani model, while in the testing phase, this improvement remained significant at 16%. In pursuit of achieving a generalized model, a dataset from meteorological stations located at various latitudes and featuring diverse climates was employed, spanning from warm-temperate regions to warm-arid zones.

Initially, only an arid climate was considered, and this methodology enabled the construction of models with a simple structure that allows for straightforward implementation. This has led us to address three main questions: Are models obtained for one region applicable to regions with slightly different conditions? Can models developed for the climate of one region be applied to another region with a different climate? For example, can a model created for an arid region be applicable to a temperate region? Is it feasible to generate a model for different climates, in this case, for arid and temperate climates, while restricting the input to measurements of temperature, relative humidity, and wind speed? To answer these questions, experiments and results were developed as described in the preceding section, demonstrating how models initially discovered for the Porvenir region in Coahuila, Mexico, were successfully applied to regions in southern California. It was also possible to identify models initially developed for an arid climate and subsequently model ETo in temperate climates. In general, models proposed in other research studies, such as those by^16,17,19^ have been developed with a specific regional focus. The need to reapply the methodology for their extension to different geographical areas underscores the limitation in their generalizability. These models, designed for particular climatic and geographical conditions, necessitate additional adjustments and validations when applied in different environments. The adaptability and transferability of these approaches to various locations and climatic contexts require careful consideration of environmental variations and the potential need for methodological adjustments to ensure accurate and effective application.

Finally, to validate the performance of the generalized model, four datasets from different latitudes with climatic characteristics similar to those used in the model development process (warm-temperate, warm-arid) were employed. The results obtained in the validation phase clearly highlight the superiority of our model designated as ETo11 over the Hargreaves–Samani model, achieving an increase of 51% in warm-temperate climates. In the case of the dataset associated with warm-arid climates, our model continued to exhibit satisfactory results by surpassing the Hargreaves–Samani model by 8%. These robust results underscore the effectiveness and versatility of our methodological approach, consolidating its suitability for addressing climatic variability and geographic heterogeneity in ETo estimation. It is important to note that in this study, the Penman‒Monteith formulation has been taken as the reference standard due to its adoption by the FAO. This standard was developed using the definition of the reference crop, which is a hypothetical crop with an assumed height of 12 cm, a surface resistance of 70 s m^−1^, and an albedo of 0.23, representing ETo from an extensively covered surface of actively growing, uniformly tall green grass that is adequately irrigated^21^. However, in dry and desert regions where local environments experience aridity effects due to insufficient ETo, the FAO56-PM model tends to overestimate ETo. A promising approach is the use of boundary layer theory in meteorological data conditioning, which allows quantification of the effects of aridity on the surface^38^. Nonetheless, its implementation poses challenges due to its complexity. The intricacy of the meteorological data conditioning method makes it impractical for widespread use in practical applications. Nevertheless, the methodology proposed in this study can easily be adapted to other reference metrics, such as measurements from a precision instrument or any other estimations.

Various studies^10,13,14,19,20^ have explored the use of machine learning models and methods such as GP to estimate ETo. The effectiveness of these approaches has been highlighted, but concerns have been raised about the inclusion of variables like solar radiation and the limitation to a single climate type, impacting the generalization of results. “Black-box” models have also shown strong but limited interpretability in their outcomes. In our case, we have developed a model addressing these concerns by training and validating it with data from two different climates (arid-warm and warm-temperate), enhancing its generalization capacity. Moreover, by employing GP as a “white-box” approach, we achieved greater transparency and understanding of key variables influencing ET0. This analytical focus improves result interpretation and underscores the importance of considering climatic diversity for a more robust and applicable model in various environmental conditions.

On the other hand, from the point of view of^39^ the annual ETo at the global level is highly correlated with solar radiation and the average annual temperature, especially when considering power or exponential regression functions. Annual ETo exhibits the expected negative correlation with average relative humidity, while it appears to be uncorrelated with wind speed. As solar radiation and temperature increase, the variability of ETo also increases significantly. It is recommended to consider at least two explanatory variables to reduce the effects of heteroscedasticity in the empirical modeling of ETo. However, GP stands out for its effectiveness in solving complex and nonlinear problems that prove challenging to model using traditional approaches. The capability of GP to explore extensive search spaces, coupled with an adaptable representation of solutions in the form of trees or graphs, enables it to address the intrinsic complexity of systems characterized by multiple variables and nonlinear relationships. In our research, we aimed to use commonly measurable meteorological parameters, opting to forego the measurement of solar radiation due to the costly equipment required for its acquisition.

Currently, the methodology has been tested using data representing specific climates. However, in our ongoing effort to enhance and broaden its applicability, we are considering the implementation of the methodology in a wider range of climates, aiming to achieve a more robust generalization. This step towards climatic diversification will allow us to assess and validate the effectiveness of the methodology in diverse environments, encompassing a variety of atmospheric and geographic conditions. This approach will not only extend the utility of the methodology but also reinforce its validity and applicability in a broader context, ensuring effective use in various locations and climatic scenarios. This process of expansion into diverse climates is regarded as a key direction for the future development of the methodology, aiming for a more solid and widespread application.

## Conclusions and future work

Accurate estimation of ETo is essential for calculating irrigation requirements and, overall, for water resource management. GP represents a powerful tool capable of developing new models to accurately estimate ETo.

This study presents an evolutionary approach using GP techniques to develop an explicit model to estimate Eto. The proposed model uses the daily climatic parameters of relative humidity, temperature, and wind speed obtained from CIMIS stations and the El Porvenir farm. The model also takes advantage of the expert knowledge provided by the FAO56-PM model. The results surpassed those of the Hargreaves–Samani model in the testing and validation stages. GP techniques have been shown to be a good tool for hydrological studies and can serve as a robust approach that can open a new field for the development of explicit formulations for many hydrological problems.

The model obtained can be used in arid-warm and warm-temperate climates and can be an alternative to the FAO-56-PM reference model in regions where only relative humidity, air temperature, and wind speed data are available. This methodology can be applied by taking other types of climates into account or limiting it to specific hydrological basins.

In future work, the consideration is to work with lysimeter data from arid areas with the aim of developing more accurate models based on measurements rather than estimations, aiming for broader model generalization. On the other hand, there is a plan to expand the data sample for training and validation to include 10, 15, and 20 years of data.

On the other hand, specific research will be conducted in sub-tropical climates, as they pose a significant challenge for ETo models. Choosing to work in this specific climatic context will allow us to test the robustness and applicability of the developed model under more complex and variable conditions. This approach will contribute to a more comprehensive understanding of the model’s effectiveness, enhancing its generalization capability and utility across diverse climatic conditions.

## Data Availability

The datasets analyzed during the current study are available upon registration in the California Irrigation Management Information System (CIMIS) repository, https://cimis.water.ca.gov/.

## Code availability

Not applicable for that section.
